# DDIT4 Novel Mutations in Pancreatic Cancer

**DOI:** 10.1155/2021/6674404

**Published:** 2021-04-30

**Authors:** Fadian Ding, Xiaoping Hong, Xiangqun Fan, Shirong Huang, Wei Lian, Xingting Chen, Qicai Liu, Youting Chen, Feng Gao

**Affiliations:** ^1^Department of Surgery, 1st Affiliated Hospital, Fujian Medical University, 350004, China; ^2^Department of Ophthalmology, 1st Affiliated Hospital, Fujian Medical University, Fuzhou 350005, China; ^3^Center of Prenatal Screening, Fujian Provincial Maternity and Children's Hospital, Fujian Medical University, Fuzhou 350005, China; ^4^Department of Laboratory Medicine, Fujian Medical University, Fuzhou 350004, China; ^5^Center for Reproductive Medicine, 1st Affiliated Hospital, Fujian Medical University, Fuzhou 350004, China; ^6^Department of Pathology, 1st Affiliated Hospital, Fujian Medical University, 20 Chazhong Road, Fuzhou 350005, China

## Abstract

Pancreatic cancer is one of the most common malignancies worldwide. This study is aimed at searching the possible genetic mutations and the value of novel gene mutation in the DNA damage-inducible transcript 4 (DDIT4) and signaling pathway in pancreatic cancer. Polymerase chain reaction (PCR) was performed to amplify the DNA sequences of DDIT4 from patients with pancreatic ductal adenocarcinoma. In addition, we used IHC to detect the expression level of DDIT4 in patients with pancreatic cancer in different types of gene mutation. Double-labeled immunofluorescence was employed to explore the expression levels of DDIT4/LC3 and their potential correlation. Our work indicated the two novel stable gene mutations in DDIT4 mRNA 3′-untranslated region (m.990 U>A and m.1246 C>U). Thirteen samples were found to have mutation in the DDIT4 3′-untranslated regions (UTR). To further verify the influence of gene mutation on protein expression, we performed immunohistochemistry on different gene mutation types, and we found a correlation between DDIT4 expression and gene mutation, which is accompanied by nuclear staining deepening. In order to further discuss the clinical value of DDIT4 gene mutation, immunofluorescence suggested that the expression of DDIT4 colocated with LC3; thus, we speculated that DDIT4 mutation may be involved in autophagy in pancreatic cancer cell. In this study, we found mutation in the 3′-UTR region of DDIT4, which may be associated with DDIT4 expression and tumor autophagy in pancreatic cancer tissues.

## 1. Introduction

Pancreatic cancer ranked the fourth most common cause of fatalities due to cancer in America. The 5-year relative survival rate of patients with pancreatic cancer was only 9% [1]. According to the research of the World Health Organization (WHO), the observed number of deaths due to pancreatic cancer was 82901 in 2014 in Europe [2]. Despite advances in conventional therapies, little improvement has been observed in the survival rate over the past 30 years [3]. Many patients with pancreatic ductal adenocarcinoma (PDAC) are either intrinsically resistant or develop acquired resistance to radiotherapy [4]. SMAD4 mutation rendered pancreatic cancer resistance to radiotherapy through promotion of autophagy [5]. However, the underlying role of gene mutation has not been fully elucidated and the mutation of autophagy-related gene will be a great treasure.

The somatic and germline genetic mutation contributes to the occurrence of pancreatic cancer, and the special gene is a novel target to kill the cancer cell. Wood et al. summarized the 24 sporadic gene mutations including the common gene mutation (KRAS, CDKN2A, TP53, SMAD4, GNAS, and RNF43) which is more than 20% prevalence [6]. Gene mutation involved in the resistance to radiotherapy in patients with pancreatic cancer [5] and the autophagy is associated with the adaptation to harsh microenvironment [7]. Combined with our previous RNA high-throughput sequencing, which showed the significant upexpression of autophagy-related protein, DDIT4 is a novel protein which is unknown in the field of genetic mutation.

DNA damage-inducible transcript 4 (DDIT4) was induced by a variety of stress conditions, including oxidative stress [8], endoplasmic reticulum stress [2], and hypoxia [6]. DDIT4 inhibited mammalian target of rapamycin complex 1 by stabilizing the tuberous sclerosis complex 2 [9]. DDIT4 was involved in the survival-related autophagy of cancer cell and affected targeted therapy for lung cancer [10]. DDIT4 was also connected to both autophagy and stemness, which were involved in temozolomide drug resistance and poor prognosis of glioblastoma multiforme patients [11]. The high level of DDIT4/TXNIP prooxidant complex regulated the ROS and inhibited the activity of ATG4B to control stress-induced autophagy [12]. So under the selection of harsh tumor microenvironment, the survival ability of pancreatic cancer cell with a certain type of gene mutation is an interesting question.

We speculated that DDIT4 gene mutation may be involved in pancreatic cancer cell adapted to antitumor therapy and harsh microenvironment. Mechanistically, the gene mutation and expression level of DDIT4 may affect the formation of autophagosomes, which will contribute to the survival of PDAC.

## 2. Methods

### 2.1. Patient Recruitment and Data Collection

Patients with pancreatic neoplasm from the First Affiliated Hospital of Fujian Medical University were included between 2018 and 2019. The patients we included were set as the experimental group and the control group, respectively, according to the pathological results. The experimental group is the group of patients with pathologically confirmed PDAC diagnosis. The control group is the group of patients with pancreatic neoplasm without PDAC. The inclusion criteria are as follows. (1) The patients had complete clinical data and follow-up data. (2) The patients signed informed consent. Patients with any comorbid or previous malignancies were excluded from the study. The related information of patients with pancreatic cancer was collected including age, gender, TMN stage, surgical method, outcome, chemotherapy regimens, pathological grading, imaging features, and the level of tumor markers (Table 1). This study was approved by the Ethics and Research Committee of the First Affiliated Hospital of Fujian Medical University.

### 2.2. Genotype Analysis

Pancreatic neoplasm tissue and adjacent healthy tissue samples were collected in liquid nitrogen and stored at -80°C. A TIANamp tissue DNA extraction kit (Tiangen Biotechnology Co., LTD.) was used to extract and store DNA from pancreatic cancer tissues and control tissues. The full-length DDIT4 was amplified and underwent gel electrophoresis following purification. Multiple sequencing was performed and analyzed by Sangon Biotech Co., Ltd. The forward primer of PCR was 5′-GTTCGCACACCCATTCAA-3′, and the reverse one was 5′-GCATAGGTCTTAATACTTGAACAT-3′. The condition of PCR was initial denaturing step, and an ABI PRISM 7700 sequencer (PerkinElmer, Inc.) was used for gene sequencing.

### 2.3. Immunohistochemistry and H&E Staining

Pancreatic tissues from patients with pancreatic cancer were used for hematoxylin and eosin staining and immunohistochemistry. DDIT4 antibodies were obtained from Arigo Biolaboratories (Hsinchu, Taiwan). Eight-micrometer sections were made in all paraffin-embedded pancreatic tissue. The slides were immunolabeled with monoclonal antibodies against DDIT4 (DNA damage-inducible transcript 4). Immunohistochemistry was performed using an automated immunohistochemical stainer according to the manufacturer's guidelines. Substitution of the primary antibody with phosphate-buffered saline was used as a negative control.

### 2.4. Immunofluorescence

Pancreatic tissues from patients were fixed with 4% paraformaldehyde, and pathological section was made. The sections were permeabilized, and nonspecific binding was blocked. Primary monoclonal antibodies specific for LC3 and DDIT4 were incubated and secondary antibodies were applied for 2 hours at room temperature, and the nucleus was stained with DAPI. The stained samples were photographed with a fluorescence inverted microscope in half an hour. Our immunofluorescence imaging uses the same exposure time.

### 2.5. Statistics

Statistical differences between groups were assessed by the nonparametric Mann–Whitney *U* test for two groups and Kruskal-Wallis test for more than two groups. Spearman's rank correlation coefficient estimated the degree of association between two variables. Significance was calculated at *P* < 0.05 by GraphPad Prism 5 (La Jolla, CA). Data are presented as mean values with standard deviation (SD) and categorical values as frequency counts and percentages. *P* < 0.05 was considered significant. All data analysis was performed using SPSS statistical software version 26.

## 3. Results

### 3.1. Two Novel Gene Mutations in DDIT4 mRNA

**Figure 1:**
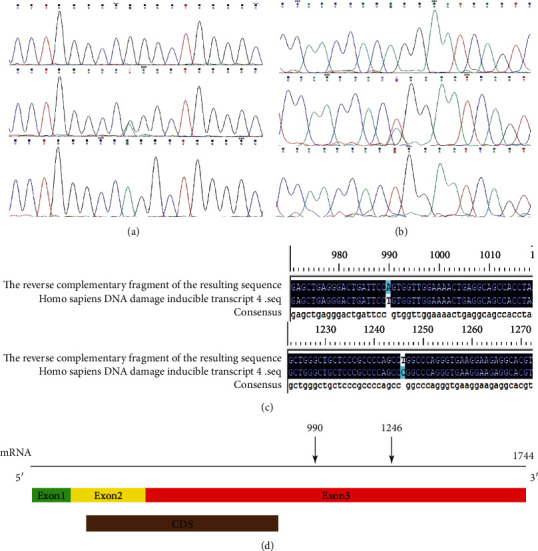
Gene mutation of DDIT4. (a, b) The reverse sequencing map gene of enrolled patients with pancreatic cancer. (c) The result of gene mutation blast match Homo sapiens DNA damage-inducible transcript 4 (DDIT4), mRNA (LOCUS: NM_019058); the blue one is the mutation point (m.990 and m.1246). (d) The two mutation sites of DDIT4 mRNA were located at exon 3 and 3′-UTR.

DDIT4 is a key molecule in the signal of autophagy, and the autophagy will prevent the cancer cell for death in the harsh microenvironment. This study used retrospective case-control study to explore the correlation analysis between gene mutation sites in the tissue of pancreatic cancer. We sequenced 15 pancreatic tissues to determine the DDIT4 gene mutation in the enrolled patients (Table 2). The best pairing sequence was Homo sapiens DNA damage-inducible transcript 4 (DDIT4), mRNA (LOCUS: NM_019058) in NCBI, which is conformed to previous experimental design. The gene mutation sites were located at m.990 U>A and m.1246 C>U, and we found three gene types at both gene sites (Figures 1(a) and 1(b)). According to the NCBI data, we found that the two mutation sites were located at DDIT4 mRNA exon 3 with a portion of 3′-UTR of gene (Figure 1(d)).

### 3.2. The Relationship between Expression Level and Protein Localization and the 3′-UTR Mutation Style of DDIT4

**Figure 2:**
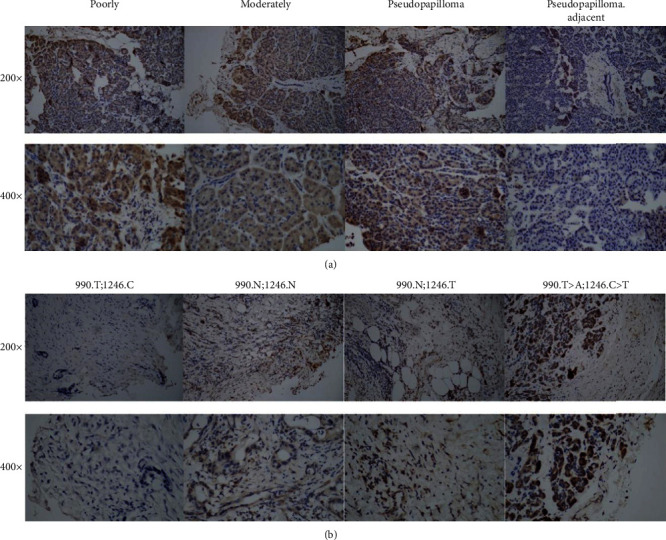
The level of DDIT4 and gene mutation/pathological grading. (a) Immunohistochemistry revealed upregulation of DDIT4 in poorly differentiated adenocarcinoma tissue, moderately differentiated adenocarcinoma tissue, and solid pseudopapilloma tissue compared with adjacent normal tissues. (b) The level of DDIT4 increased in DDIT4 3′-UTR gene mutation (990.T>A;1246.C>T) compared with pancreatic cancer tissues without gene mutation. The expression level of DDIT4 increased both in the cytoplasm and nucleus.

DDIT4 correlated with tumor progression and affected the prognosis of patients with ovarian carcinoma [13]. In order to explore the relationship between DDIT4 and the pancreatic pathological grades, we performed IHC in pancreatic tissue. Our results indicated that DDIT4 had a higher expression level in poor differentiated adenocarcinoma compared to other pancreatic cancer pathological styles including the moderately differentiated adenocarcinoma, highly differentiated adenocarcinoma, and false papilloma of pancreatic cancer. Under a ×400 field of view, the level of DDIT4 significantly increased both in the nucleus and cytoplasm. The underlying mechanism of high DDIT4 expression in poorly differentiated adenocarcinoma may be related to mutation (Figure 2(a)). In order to further clarify the correlation between 3′-UTR mutation and DDIT4 expression level, we performed IHC in pancreatic tumor tissue including several combinations of two types of genetic mutations. And then, we set up four groups (group 1: 990.T;1246.C; group 2: 990.N;1246.N; group 3: 990.N;1246.T; and group 4: 990.T>A;1246.C>T), and our result suggested that group 4 had the highest expression level (Figure 2(b)), followed by group 2 and group 3, while group 4 had the lowest expression level. Through the analysis of the results, we believed that the two DDIT4 3′-UTR gene mutations may be involved in the protein expression level. Under a ×400 field of view, DDIT4 was mainly located in the cytoplasm while it deepened in the nucleus.

### 3.3. The Activation of DDIT4/LC3 Signaling Pathways in Pancreatic Cancer

**Figure 3:**
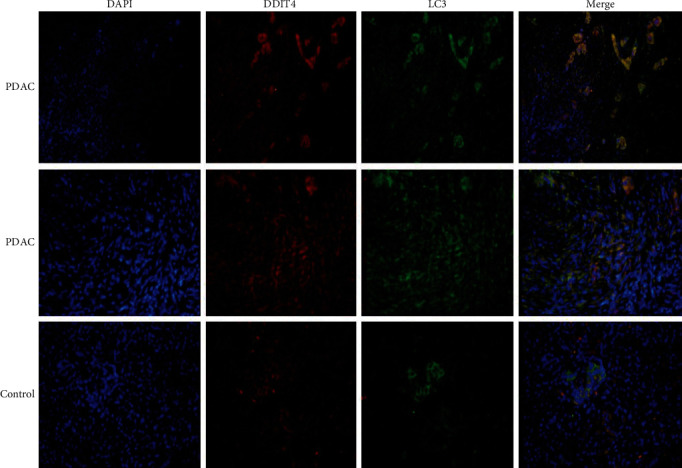
Fluorescence showed the distributions of DDIT4 and LC3 in pancreatic cancer tissue.

Previous literatures suggested that DDIT4 was closely related to autophagy in tumor cells [11]. To further clarify the correlation between DDIT4 expression level and autophagy in pancreatic cancer tissues, we performed immunofluorescence on pancreatic cancer tissues which confirmed that the expression level of LC3 and DDIT4 was significantly high in pancreatic cancer at the same time compared to the control (the low expression level of DDIT4). And we found that the expression of LC3 and DDIT4 was synchronized (Figure 3).

### 3.4. The Perspective View of DDIT4 in Pancreatic Cancer

**Figure 4:**
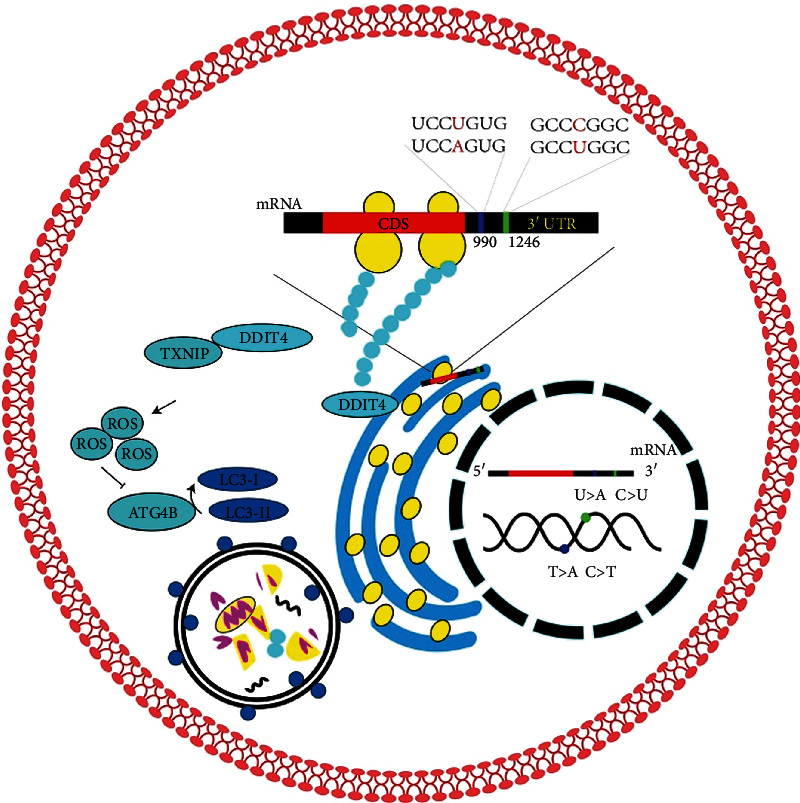
Hypothetical image of DDIT4 mRNA mutation in pancreatic cancer.

Based on our experimental results and relevant literature, we summarized the relationship between DDIT4 3′-UTR mutation and pancreatic cancer. The DDIT4 gene mutation in pancreatic cancer cells leads to a base-point mutation in DDIT4 3′-UTR, and it may affect protein localization which may be involved in its biological function. The DDIT4/TXNIP complex located in the mitochondrion promotes the expression of ROS, which inhibited the dephosphorylation ability of ATG4B. And it promotes the increased amount of LC3 expression and the activation of autophagy. DDIT4 gene mutations may result in the antitumor resistance through abnormal protein localization in the pancreatic tumor cells (Figure 4).

## 4. Discussion

Our article found two novel gene mutations in the 3′-untranslated region of DDIT4 mRNA in PDAC. At the same time, we found a correlation between the expression level of DDIT4 and the type of gene mutation, and DDIT4 was colocated with LC3 obviously in pancreatic cancer. This paper showed that the two novel gene mutations in the 3′-untranslated region of DDIT4 mRNA may be a novel target in pancreatic cancer.

We found two types of stable mutation in the DDIT4 mRNA 3′-UTR, and the most prevalent class of regulatory elements in the 3′-UTR is microRNA binding sites [14]. A gene mutation that resulted in a premature polyadenylation signal in CCND1 shortened its 3′-UTR and increased the risk of lymphoma [15]. Combined with our results, microRNA may be closely related to DDIT4 function in pancreatic cancer. Previous literatures revealed that miR-221 contributed to the hepatocarcinogenesis via the dysregulation of DDIT4 [16]. At the same time, miR-221 played an important role in human placental development by precisely regulating the DDIT4 expression [17]. There were some other miRNA involved in the expression of DDIT4, such as miR-495 [18], miR-30c [19], and miR-630 [20]. In summary, DDIT4 has gene mutations in 3′-UTR in pancreatic cancer tissues, which may be related to the binding site of microRNA and affected the expression level and localization of DDIT4.

The expression levels of DDIT4 were regulated by a variety of factors, such as hypoxia [21], ionizing radiation [22], energy depletion [23], and endoplasmic reticulum stress [24]. Our results were consistent with previous study about the relationship between pathological grade and protein level in ovarian carcinoma [13]. It is possible that the poor differentiated malignancy was associated with higher rate of gene mutation. Our paper found the correlation between DDIT4 3′-UTR gene mutation and DDIT4. Mutation in genes was the most important feature in tumor, and accumulation of genetic mutation will lead to abnormal behavior of cancer cells [25]. DDIT4 3′-UTR gene mutation may affect protein localization and protein degradation by affecting the binding of DDIT4 to microRNA.

The colocalization of DDIT4 and LC3 indicated that DDIT4 was highly expressed in pancreatic cancer tissues and its expression is involved in autophagy. DDIT4 signaling was connected to both autophagy which were involved in temozolomide drug resistance and the poor prognoses of glioblastoma multiforme patients [11]. The ATF4-DDIT4-mTORC1 axis will inhibit the cancer therapy by activation of the autophagy signal pathway, and the combinatorial treatment with SIRT1/2 inhibitors and pharmacological autophagy inhibitors was an effective therapeutic strategy in lung cancer [10]. Gene mutation in DDIT4 3′-UTR will affect the autophagy by regulating the expression level of DDIT4.

The high expression of DDIT4 not only promotes the increase of autophagy in pancreatic cancer tumor cells but also may be involved in tumor resistance by inhibiting the function of immune cells around the tumor [26]. Meanwhile, the abnormal function of immune cells was involved in tumor resistance of pancreatic cancer. DDIT4 enhanced vascular inflammation and permeability in endotoxemia mice, leading to immune cell infiltration, systemic inflammation, caspase-3 activation, and apoptosis [27]. DDIT4 was related to the low level of reactive oxygen species of mitochondria in macrophages induced by IL-10 or hypoxia [28], and it inhibited the immune function of macrophages. Mast cell-activated cancer-associated fibroblasts (CAFs) and transforming growth factor-*β* signaling were involved in pancreatic cancer resistance to gemcitabine/nabaxel [29]. A therapeutic strategy targeting Wnt enhanced pancreatic cancer cytotoxicity and restored anticancer immunity in patients with nodular positive disease [30].

The heterogeneity of cancer is ultimately related to drug resistance after long-term codevelopment of tumor and microenvironment, and the mutation of drug-resistant genes is the inevitable result which benefits the survival of tumor. The UTR mutation of DDIT4 is involved in autophagy of pancreatic cancer cells by regulating the expression of DDIT4, and it may be a potential biomarker for chemotherapy resistance and poor prognosis. The overexpression of ADAM28 in pancreatic cancer is closely related to the regulation of gemcitabine resistance, so it is a new prognostic biomarker in pancreatic cancer [31]. High expression of miR-155-5p is directly associated with chemotherapy resistance and poor prognosis in PDAC patients treated with gemcitabine [32]. The mutation of autophagy-related gene DDIT4 and the higher protein expression level are of great significance. In the future, we may monitor the mutation level of DDIT4 in tissues through detecting the mutation level of DDIT4 in blood samples of pancreatic cancer patients and then realize effective assessments of chemotherapy resistance and poor prognosis.

This article has some limitations. The survival time of patients with pancreatic cancer is very short, and it is difficult to use cohort study. Therefore, this study used retrospective case-control study to explore the correlation analysis between gene mutation sites and tumor self-dependence and to provide a new target for chemotherapy resistance and prognosis in the future. This article only captured the tumor tissue at a point in time and through the comprehensive analysis of the DDIT4 gene mutations in multiple tumor samples, but the evidence is indirect. In the future, if we are lucky enough to get the same tumor patients with multiple points in time of tissue or blood samples, we can directly observe the gene mutation during the progression of the tumor.

## 5. Conclusion

In this study, we found a gene mutation in the 3′-UTR region of DDIT4, which may be associated with DDIT4 expression and tumor autophagy in pancreatic cancer tissues, and the further mechanistic research requires more work.

## Figures and Tables

**Table 1:** Basic clinical characteristics of patients.

No.	Gender	Stages	Surgical method	Outcome	Chemotherapy regimens	Pathology	Image features	Tumor markers
1	Female	IIB (pT2N1M0)	Radical pancreaticoduodenectomy	Adjuvant therapy	(Gemcitabine 1.4 g D1, 8, 15 q4w)∗1 cycle	Moderately differentiated adenocarcinoma	2.5∗2.5∗2 cm	HER2(0), P53(70%+), MUC6(-), MUC2(-), MUC1(+), CDX2(-), MUC5A(+), SATB2(-), CK20(-), CK7(+), CK19(+), CD34(+)
2	Female	IB (pT2N0M0)	Partial pancreatectomy with duodenal reservation	Adjuvant therapy	Unknown	Solid pseudopapilloma	3.2∗3.0∗1.8 cm	*β*-Catenin(+), vim(+), CK(-), CD10(+), syn(-), EMA(-), CEA(+), CDX2(-), AFP(-), CK(-), CK8/18(-), WT-1(-), MUC1(-), MUC5A(-)
3	Male	IIA (T3N0M0)	Radical pancreaticoduodenectomy	Liver metastasis	(Gemcitabine 0.8 g D1, D8 + cisplatin 30 mg D1‐2, q3w)∗2 cycles; (albumin paclitaxel 200 mg q2w)∗2 cycles	Moderately differentiated adenocarcinoma	2.5∗2.4∗2 cm	P53(+), HER2(1+), Ki-67(80%+), MUC1(+), MUC2(-), MUC5(-), MUC6(-)
4	Female	IIIA (pT4N0M0)	Partial pancreatectomy	Multiple abdominal metastases (lymph node and abdominal wall)	Did not receive chemotherapy because general condition is bad	Moderately differentiated adenocarcinoma	3.5∗3∗2 cm	MUC1(+), MUC2(-), MUC5(+), MUC6(-), CK7(+), CK20(+), CDX2(+), Ki-67(+60%), P53(+70%), HER2(+)
5	Male	IIIA (pT4N0M0)	Partial pancreatectomy	Septic shock and death	Vacant	Moderately differentiated adenocarcinoma	1.5∗1∗0.7 cm	Vacant
6	Male	III (pT1N2M0)	Partial pancreatectomy with duodenal reservation	Local recurrence and liver metastasis	(Gemcitabine 1.4 g D1, D8 + oxaliplatin 150 mg D1)∗1 cycle; (gemcitabine 1.4 mg D1, D8 + tegafur 3#qm, 2#qn D1‐D14)∗2 cycles	Moderately differentiated adenocarcinoma	1.5∗0.8∗0.4 cm	CK7(+), MUC5(+), MUC1(+), CK20(-), MUC2(-), CK8/18(+), CK19(+), EMA(+), CA199(+), Ki-67(+10%)
7	Male	IV (pT2n1m1)	Radical pancreaticoduodenectomy	Adjuvant therapy	(Capecitabine 1.5 g bid D1‐14 + gemcitabine 1.4 g D 1, 8, q3w)∗8 cycles	Moderately differentiated adenocarcinoma	5.5∗4.0∗3.0 cm	MUC1(+), CK7(+), MUC5(+), CK20(+), SMAD4(+), MUC2(-), P53(+80%), HER2(1+), Ki-67(+40%), CDX2(+)
8	Female	IIB (pT3N1M0)	Radical pancreaticoduodenectomy	Adjuvant therapy	(Gemcitabine 1.6 g D1, D8 + capecitabine 2 g D2‐D15)∗6 cycles mFOLFIRINOX∗4 cycles (Gemcitabine 1.4 g + albumin paclitaxel 200 mg)∗1 cycle	Poorly differentiated adenocarcinoma	3∗3∗2.5 cm	CK20(+), CK7(+), MUC5(+), MUC1(+), CK818(+), CK19(+), EMA(+), CA199(+), Ki-67(10%), MUC2(-), D2-40(+), CD34(+)

**Table 2:** Relationship between DDIT4 mutation and pancreatic cancer.

*N*	Gender	Tissue types	Pathologic types	mRNA.990	mRNA.1246
1	Female	Cancerous	Moderately differentiated adenocarcinoma	T>A	C>T
2	Female	Paracancerous	—	T>A	C>T
3	Female	Cancerous	Solid pseudopapilloma	N	N
4	Female	Paracancerous	—	N	N
5	Male	Cancerous	Moderately differentiated adenocarcinoma	N	N
6	Male	Paracancerous	—	N	N
7	Female	Cancerous	Moderately differentiated adenocarcinoma	T>A	C>T
8	Female	Paracancerous	—	T>A	C>T
9	Male	Cancerous	Moderately differentiated adenocarcinoma	N	C>T
10	Male	Paracancerous	—	N	C>T
11	Male	Cancerous	Poorly differentiated adenocarcinoma	T	C
12	Male	Paracancerous	—	T	C
13	Male	Cancerous	Poorly differentiated adenocarcinoma	N	C>T
14	Male	Paracancerous	—	N	C>T
15	Female	Cancerous	Poorly differentiated adenocarcinoma	T	N

## Data Availability

Data is available upon request from the authors.
